# 4-Des­oxy-4β-[(5-meth­oxy-1*H*-indol-3-yl)oxalylamino]podophyllotoxin methanol solvate

**DOI:** 10.1107/S1600536808035319

**Published:** 2008-11-13

**Authors:** Min Feng, Ming Zhao, Jingze Zhang, Zaixin Yang, Hong Chen

**Affiliations:** a School of Pharmaceutical Sciences, Tianjin Medical University, Tianjin 300071, People’s Republic of China; b Room of Pharmacognosy, Medical College of Chinese People’s Armed Police Forces, Tianjin 300162, People’s Republic of China

## Abstract

The main mol­ecule of the title solvate, C_33_H_30_N_2_O_10_·CH_3_OH, is a new anti­tumor agent, which shows cytotoxicity against MDR cancer cell lines. It has been synthesized by coupling 4β-amino­podophyllotoxin with (5-meth­oxy-1*H*-indol-3-yl)glyoxyl chloride and structurally characterized. There are two crystallographically independent mol­ecules in the asymmetric unit, which differ in the dihedral angles between the aromatic rings. The dihedral angles between the benzene ring of the benzo[*d*][1,3]dioxole and the benzene ring of the 5-meth­oxy-1*H*-indole are 85.08 (3) and 76.88 (3)° and reflect the main conformational difference between the two independent mol­ecules. The asymmetric unit is completed with two methanol solvent mol­ecules, one of which is disordered over two positions, with occupancies close to 0.5.

## Related literature

For related preparation and anti­tumor activity, see: Yu *et al.* (2008 ▶); Knaack *et al.* (2001 ▶); Zhou *et al.* (1991 ▶); Zhu *et al.* (1999 ▶); Chen *et al.* (2006 ▶).
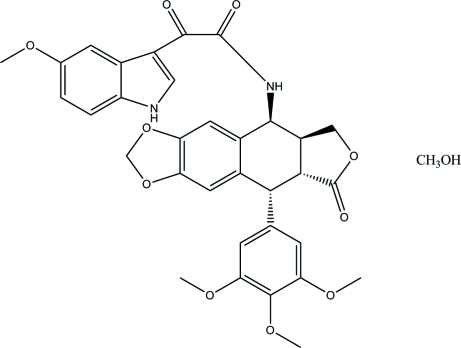


## Experimental

### 

#### Crystal data

C_33_H_30_N_2_O_10_·CH_4_O
*M* *_r_* = 646.63
Monoclinic, 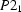

*a* = 12.228 (2) Å
*b* = 9.8360 (16) Å
*c* = 26.667 (5) Å
β = 95.58 (3)°
*V* = 3192.2 (9) Å^3^
*Z* = 4
Mo *K*α radiation
μ = 0.10 mm^−1^
*T* = 113 (2) K
0.24 × 0.22 × 0.12 mm

#### Data collection

Rigaku Saturn CCD area-detector diffractometer
Absorption correction: multi-scan (*CrystalClear*; Rigaku/MSC, 2005 ▶) *T*_min_ = 0.976, *T*_max_ = 0.988
22853 measured reflections
7730 independent reflections
6754 reflections with *I* > 2σ(*I*)
*R*_int_ = 0.045

#### Refinement

*R*[*F* ^2^ > 2σ(*F* ^2^)] = 0.047
*wR*(*F* ^2^) = 0.127
*S* = 1.03
7730 reflections
893 parameters
H atoms treated by a mixture of independent and constrained refinement
Δρ_max_ = 0.73 e Å^−3^
Δρ_min_ = −0.27 e Å^−3^

### 

Data collection: *CrystalClear* (Rigaku/MSC, 2005 ▶); cell refinement: *CrystalClear*; data reduction: *CrystalClear*; program(s) used to solve structure: *SHELXS97* (Sheldrick, 2008 ▶); program(s) used to refine structure: *SHELXL97* (Sheldrick, 2008 ▶); molecular graphics: *SHELXTL* (Sheldrick, 2008 ▶); software used to prepare material for publication: *SHELXTL*.

## Supplementary Material

Crystal structure: contains datablocks global, I. DOI: 10.1107/S1600536808035319/bh2197sup1.cif

Structure factors: contains datablocks I. DOI: 10.1107/S1600536808035319/bh2197Isup2.hkl

Additional supplementary materials:  crystallographic information; 3D view; checkCIF report

## Figures and Tables

**Fig. 1.**
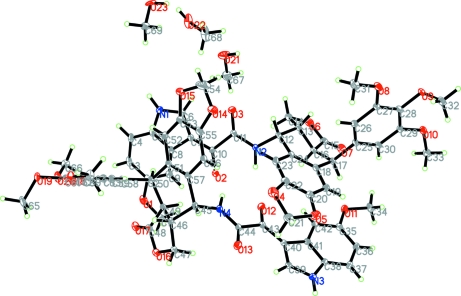
The molecular structure of the main molecules, with atom labels and 50% probability displacement ellipsoids for non-H atoms. The crystal is a methanol solvate, with a 1:1 composition with respect to the main molecule. Solvent molecules were omitted.

## supplementary crystallographic information

### Comment

Podophyllotoxins are a particularly instructive class of compounds for consideration in the design and synthesis of potential anticancer agents based upon natural products prototypes. We present here the crystal structure of the title compound (Fig. 1), which can be used as a precursor for antitumor molecules. In the crystal structure, two crystallographically independent molecules are found in the asymmetric unit. The mean planes for the three benzene rings of benzo[*d*][1,3]dioxole (ring A), 3,4,5-trimethoxyphenyl (ring B) and 5-methoxy-1*H*-indole (ring C), form dihedral angles A/B = 88.00 (3) and 83.26 (3)°, B/C = 44.66 (3) and 50.01 (4)°, and A/C = 85.08 (3)/76.88 (3)°, respectively. This makes clear that the main difference between conformations in both independent molecules arises from A and C rings (dihedral angles differ by more than 8°), while the podophyllotoxin core structure remains more or less rigid.

### Experimental

4β-Aminopodophyllotoxin (1.0 mmol), 5-methoxy-indol-3-yl-glyoxyl chloride (1.0 mmol) and Et_3_N (2.0 mmol) were dissolved in dry dichloromethane. The mixture was stirred at room temperature for 36 h., and then extracted with dichloromethane after 30 ml water was added. The organic phase was dried over Na_2_SO_4_ and concentrated *in vacuo*. The residue was purified by column chromatography on silica gel using petroleum ether/ethyl acetate as eluent. Single crystals suitable for X-ray diffraction were prepared by evaporation of a methanol solution of the product.

### Refinement

In absence of significant anomalous dispersion effects, Friedel pairs were merged. The absolute configuration was assigned by reference to the known configuration of the starting material. One methanol molecule is disordered over two positions, and corresponding site occupation factors were refined to 0.522 (5) and 0.478 (5). All H atoms of N—H groups were located in a difference map and refined freely. All H atoms for O—H groups were placed in idealized positions, with O—H bond lengths of 0.84 Å. Other H atoms were positioned geometrically and refined using a riding model, with C—H bond lengths in the range 0.95–0.99 Å and *U*_iso_(H) = 1.2*U*_eq_(C) or 1.5*U*_eq_(C_methyl_).

### Crystal data

**Table:**

C_33_H_30_N_2_O_10_·CH_4_O	*F*_000_ = 1360
*M**_r_* = 646.63	*D*_x_ = 1.345 Mg m^−^^3^
Monoclinic, *P*2_1_	Mo *K*α radiation λ = 0.71073 Å
Hall symbol: P 2yb	Cell parameters from 8161 reflections
*a* = 12.228 (2) Å	θ = 1.7–27.5º
*b* = 9.8360 (16) Å	µ = 0.10 mm^−^^1^
*c* = 26.667 (5) Å	*T* = 113 (2) K
β = 95.58 (3)º	Plate, yellow
*V* = 3192.2 (9) Å^3^	0.24 × 0.22 × 0.12 mm
*Z* = 4	

### Data collection

**Table:**

Rigaku Saturn CCD area-detector diffractometer	7730 independent reflections
Radiation source: rotating anode	6754 reflections with *I* > 2σ(*I*)
Monochromator: confocal	*R*_int_ = 0.045
Detector resolution: 7.31 pixels mm^-1^	θ_max_ = 27.5º
*T* = 113(2) K	θ_min_ = 1.5º
ω and φ scans	*h* = −15→15
Absorption correction: multi-scan(CrystalClear; Rigaku/MSC, 2005)	*k* = −12→11
*T*_min_ = 0.976, *T*_max_ = 0.988	*l* = −31→34
22853 measured reflections	

### Refinement

**Table:**

Refinement on *F*^2^	Hydrogen site location: inferred from neighbouring sites
Least-squares matrix: full	H atoms treated by a mixture of independent and constrained refinement
*R*[*F*^2^ > 2σ(*F*^2^)] = 0.047	*w* = 1/[σ^2^(*F*_o_^2^) + (0.0825*P*)^2^] where *P* = (*F*_o_^2^ + 2*F*_c_^2^)/3
*wR*(*F*^2^) = 0.127	(Δ/σ)_max_ = 0.001
*S* = 1.03	Δρ_max_ = 0.73 e Å^−^^3^
7730 reflections	Δρ_min_ = −0.27 e Å^−^^3^
893 parameters	Extinction correction: SHELXL97 (Sheldrick, 2008), Fc^*^=kFc[1+0.001xFc^2^λ^3^/sin(2θ)]^-1/4^
Primary atom site location: structure-invariant direct methods	Extinction coefficient: 0.0201 (14)
Secondary atom site location: difference Fourier map	

### Fractional atomic coordinates and isotropic or equivalent isotropic displacement parameters (Å^2^)

**Table:**

	*x*	*y*	*z*	*U*_iso_*/*U*_eq_	Occ. (<1)
O1	0.9709 (2)	0.2102 (3)	0.43373 (9)	0.0355 (6)	
O2	0.83051 (16)	0.5566 (2)	0.29071 (8)	0.0237 (5)	
O3	0.56989 (19)	0.5637 (3)	0.22380 (9)	0.0430 (7)	
O4	0.7776 (2)	1.1791 (3)	0.23313 (9)	0.0381 (6)	
O5	0.89806 (19)	1.2324 (3)	0.17487 (9)	0.0344 (6)	
O6	0.74319 (19)	0.4426 (2)	0.07044 (9)	0.0318 (5)	
O7	0.8771 (2)	0.5210 (2)	0.02670 (9)	0.0338 (5)	
O8	0.51000 (18)	1.0135 (3)	−0.01957 (8)	0.0303 (5)	
O9	0.59367 (17)	0.9543 (2)	−0.10683 (8)	0.0251 (5)	
O10	0.79144 (17)	0.8430 (3)	−0.10779 (8)	0.0285 (5)	
O11	1.10961 (19)	0.6741 (2)	0.06729 (8)	0.0293 (5)	
O12	0.96392 (17)	0.7624 (2)	0.23470 (8)	0.0252 (5)	
O13	1.0023 (2)	1.0455 (3)	0.31461 (8)	0.0324 (5)	
O14	0.45644 (19)	0.9379 (3)	0.28103 (9)	0.0392 (6)	
O15	0.39852 (17)	0.7404 (3)	0.31787 (9)	0.0350 (6)	
O16	1.05011 (18)	0.7382 (3)	0.45386 (8)	0.0312 (5)	
O17	0.97883 (19)	0.5355 (2)	0.47195 (8)	0.0296 (5)	
O18	0.59318 (19)	0.8892 (2)	0.55775 (8)	0.0273 (5)	
O19	0.61900 (16)	0.6673 (2)	0.61567 (7)	0.0238 (5)	
O20	0.7020 (2)	0.4377 (2)	0.58168 (8)	0.0319 (5)	
N1	0.5534 (2)	0.3107 (3)	0.34849 (10)	0.0280 (6)	
H1	0.490 (3)	0.275 (4)	0.3584 (14)	0.034*	
N2	0.7327 (2)	0.6542 (3)	0.20608 (9)	0.0229 (5)	
H2	0.812 (3)	0.671 (4)	0.2177 (13)	0.027*	
N3	1.2084 (2)	1.0934 (3)	0.19960 (10)	0.0249 (6)	
H3A	1.252 (3)	1.166 (4)	0.2089 (14)	0.030*	
N4	0.8928 (2)	0.8590 (3)	0.31837 (9)	0.0212 (5)	
H4A	0.865 (3)	0.788 (4)	0.3026 (13)	0.025*	
C1	1.0619 (3)	0.2694 (4)	0.41211 (13)	0.0358 (8)	
H1A	1.0601	0.2424	0.3767	0.054*	
H1B	1.1306	0.2379	0.4304	0.054*	
H1C	1.0576	0.3687	0.4144	0.054*	
C2	0.8683 (3)	0.2420 (3)	0.41280 (12)	0.0261 (7)	
C3	0.7839 (3)	0.1797 (3)	0.43615 (12)	0.0294 (7)	
H3	0.8015	0.1254	0.4653	0.035*	
C4	0.6750 (3)	0.1962 (3)	0.41739 (12)	0.0292 (7)	
H4	0.6173	0.1532	0.4329	0.035*	
C5	0.6535 (3)	0.2779 (3)	0.37513 (11)	0.0242 (6)	
C6	0.5696 (3)	0.3946 (3)	0.31033 (11)	0.0262 (7)	
H6	0.5134	0.4301	0.2869	0.031*	
C7	0.6824 (2)	0.4218 (3)	0.31032 (11)	0.0216 (6)	
C8	0.7367 (2)	0.3440 (3)	0.35189 (10)	0.0200 (6)	
C9	0.8474 (2)	0.3262 (3)	0.37056 (11)	0.0232 (6)	
H9	0.9052	0.3693	0.3552	0.028*	
C10	0.7340 (2)	0.5197 (3)	0.28133 (10)	0.0202 (6)	
C11	0.6682 (2)	0.5823 (3)	0.23466 (11)	0.0252 (7)	
C12	0.6859 (2)	0.7245 (3)	0.16045 (11)	0.0238 (6)	
H12	0.6060	0.7400	0.1634	0.029*	
C13	0.6968 (2)	0.6420 (3)	0.11236 (11)	0.0227 (6)	
H13	0.6432	0.6794	0.0851	0.027*	
C14	0.6835 (3)	0.4872 (4)	0.11279 (13)	0.0305 (7)	
H14A	0.7163	0.4479	0.1450	0.037*	
H14B	0.6051	0.4610	0.1075	0.037*	
C15	0.8175 (3)	0.5382 (3)	0.05986 (12)	0.0268 (7)	
C16	0.8111 (2)	0.6562 (3)	0.09543 (10)	0.0211 (6)	
H16	0.8642	0.6368	0.1255	0.025*	
C17	0.8347 (2)	0.8009 (3)	0.07850 (10)	0.0195 (6)	
H17	0.9144	0.8065	0.0732	0.023*	
C18	0.8143 (2)	0.8956 (3)	0.12187 (11)	0.0209 (6)	
C19	0.8700 (2)	1.0211 (3)	0.12473 (11)	0.0236 (6)	
H19	0.9181	1.0461	0.1004	0.028*	
C20	0.8522 (2)	1.1066 (3)	0.16423 (12)	0.0254 (6)	
C21	0.8708 (3)	1.2608 (4)	0.22494 (13)	0.0362 (8)	
H21A	0.9334	1.2381	0.2499	0.043*	
H21B	0.8531	1.3584	0.2284	0.043*	
C22	0.7809 (3)	1.0735 (3)	0.19899 (12)	0.0278 (7)	
C23	0.7237 (2)	0.9546 (4)	0.19687 (11)	0.0260 (7)	
H23	0.6733	0.9344	0.2208	0.031*	
C24	0.7416 (2)	0.8628 (3)	0.15812 (10)	0.0222 (6)	
C25	0.7684 (2)	0.8429 (3)	0.02942 (10)	0.0196 (6)	
C26	0.6673 (2)	0.9084 (3)	0.02973 (11)	0.0225 (6)	
H26	0.6383	0.9271	0.0608	0.027*	
C27	0.6086 (2)	0.9465 (3)	−0.01582 (11)	0.0225 (6)	
C28	0.6521 (2)	0.9191 (3)	−0.06129 (11)	0.0233 (6)	
C29	0.7545 (2)	0.8577 (3)	−0.06123 (11)	0.0213 (6)	
C30	0.8123 (2)	0.8184 (3)	−0.01594 (11)	0.0226 (6)	
H30	0.8816	0.7748	−0.0160	0.027*	
C31	0.4672 (3)	1.0519 (5)	0.02636 (14)	0.0418 (9)	
H31A	0.4544	0.9702	0.0460	0.063*	
H31B	0.3978	1.1008	0.0187	0.063*	
H31C	0.5202	1.1109	0.0459	0.063*	
C32	0.6137 (3)	1.0929 (4)	−0.12153 (14)	0.0344 (8)	
H32A	0.5877	1.1554	−0.0967	0.052*	
H32B	0.5744	1.1109	−0.1547	0.052*	
H32C	0.6927	1.1063	−0.1232	0.052*	
C33	0.9057 (3)	0.8143 (5)	−0.10879 (13)	0.0363 (8)	
H33A	0.9491	0.8824	−0.0888	0.054*	
H33B	0.9245	0.8170	−0.1437	0.054*	
H33C	0.9218	0.7237	−0.0946	0.054*	
C34	1.1325 (3)	0.6781 (4)	0.01564 (12)	0.0318 (7)	
H34A	1.1113	0.7670	0.0012	0.048*	
H34B	1.0906	0.6066	−0.0032	0.048*	
H34C	1.2112	0.6634	0.0136	0.048*	
C35	1.1422 (2)	0.7832 (3)	0.09760 (11)	0.0228 (6)	
C36	1.2157 (2)	0.8833 (3)	0.08386 (12)	0.0256 (6)	
H36	1.2449	0.8779	0.0522	0.031*	
C37	1.2456 (3)	0.9898 (3)	0.11633 (12)	0.0268 (7)	
H37	1.2959	1.0575	0.1076	0.032*	
C38	1.1995 (2)	0.9946 (3)	0.16239 (11)	0.0235 (6)	
C39	1.1422 (2)	1.0624 (3)	0.23537 (11)	0.0220 (6)	
H39	1.1337	1.1159	0.2644	0.026*	
C40	1.0877 (2)	0.9400 (3)	0.22359 (11)	0.0208 (6)	
C41	1.1253 (2)	0.8955 (3)	0.17615 (11)	0.0196 (6)	
C42	1.0986 (2)	0.7857 (3)	0.14365 (11)	0.0214 (6)	
H42	1.0517	0.7148	0.1530	0.026*	
C43	1.0061 (2)	0.8723 (3)	0.24936 (10)	0.0206 (6)	
C44	0.9674 (2)	0.9361 (3)	0.29783 (10)	0.0216 (6)	
C45	0.8387 (2)	0.9029 (3)	0.36257 (10)	0.0218 (6)	
H45	0.8402	1.0044	0.3644	0.026*	
C46	0.8942 (2)	0.8449 (3)	0.41195 (11)	0.0221 (6)	
H46	0.8586	0.8860	0.4406	0.027*	
C47	1.0178 (3)	0.8571 (4)	0.42313 (12)	0.0289 (7)	
H47A	1.0542	0.8567	0.3916	0.035*	
H47B	1.0375	0.9420	0.4418	0.035*	
C48	0.9684 (3)	0.6421 (3)	0.45032 (11)	0.0254 (7)	
C49	0.8766 (2)	0.6920 (3)	0.41273 (11)	0.0216 (6)	
H49	0.8944	0.6581	0.3791	0.026*	
C50	0.7568 (2)	0.6525 (3)	0.41697 (10)	0.0210 (6)	
H50	0.7486	0.5558	0.4057	0.025*	
C51	0.6837 (2)	0.7376 (3)	0.37934 (10)	0.0208 (6)	
C52	0.5740 (2)	0.6926 (3)	0.36810 (11)	0.0235 (6)	
H52	0.5484	0.6129	0.3834	0.028*	
C53	0.5061 (2)	0.7661 (3)	0.33488 (12)	0.0257 (7)	
C54	0.3728 (4)	0.8367 (6)	0.2790 (2)	0.0734 (17)	
H54A	0.3010	0.8796	0.2832	0.088*	
H54B	0.3673	0.7906	0.2458	0.088*	
C55	0.5417 (3)	0.8846 (4)	0.31276 (12)	0.0284 (7)	
C56	0.6461 (2)	0.9325 (3)	0.32251 (11)	0.0247 (6)	
H56	0.6691	1.0140	0.3076	0.030*	
C57	0.7191 (2)	0.8550 (3)	0.35611 (10)	0.0203 (6)	
C58	0.7206 (2)	0.6588 (3)	0.47014 (11)	0.0202 (6)	
C59	0.6749 (2)	0.7768 (3)	0.48856 (11)	0.0216 (6)	
H59	0.6664	0.8554	0.4679	0.026*	
C60	0.6418 (2)	0.7796 (3)	0.53700 (11)	0.0197 (6)	
C61	0.6544 (2)	0.6648 (3)	0.56788 (11)	0.0207 (6)	
C62	0.6964 (2)	0.5457 (3)	0.54896 (11)	0.0212 (6)	
C63	0.7300 (2)	0.5425 (3)	0.50054 (11)	0.0235 (6)	
H63	0.7595	0.4612	0.4881	0.028*	
C64	0.5741 (3)	1.0045 (3)	0.52584 (13)	0.0303 (7)	
H64A	0.6446	1.0426	0.5182	0.045*	
H64B	0.5331	1.0732	0.5429	0.045*	
H64C	0.5315	0.9770	0.4945	0.045*	
C65	0.7057 (3)	0.6693 (6)	0.65580 (12)	0.0495 (12)	
H65A	0.7580	0.5963	0.6508	0.074*	
H65B	0.6748	0.6559	0.6880	0.074*	
H65C	0.7435	0.7572	0.6561	0.074*	
C66	0.6889 (3)	0.3055 (3)	0.55972 (13)	0.0300 (7)	
H66A	0.6229	0.3040	0.5358	0.045*	
H66B	0.6816	0.2382	0.5863	0.045*	
H66C	0.7534	0.2837	0.5421	0.045*	
O21	0.3925 (2)	0.2634 (4)	0.18772 (15)	0.0800 (13)	
H21	0.3869	0.3251	0.1659	0.120*	
C67	0.4991 (4)	0.2072 (5)	0.19056 (18)	0.0584 (13)	
H67A	0.5536	0.2796	0.1974	0.088*	
H67B	0.5103	0.1631	0.1585	0.088*	
H67C	0.5075	0.1399	0.2178	0.088*	
O22	0.1884 (6)	0.3747 (4)	0.24506 (19)	0.0478 (17)	0.522 (5)
H22	0.1301	0.3932	0.2579	0.072*	0.522 (5)
C68	0.2343 (10)	0.4948 (7)	0.2278 (3)	0.065 (3)	0.522 (5)
H68A	0.1854	0.5715	0.2328	0.097*	0.522 (5)
H68B	0.2437	0.4859	0.1919	0.097*	0.522 (5)
H68C	0.3060	0.5108	0.2467	0.097*	0.522 (5)
O23	0.0596 (3)	0.4971 (5)	0.3106 (2)	0.0326 (13)	0.478 (5)
H23A	0.0792	0.4853	0.2816	0.049*	0.478 (5)
C69	0.1315 (5)	0.5923 (8)	0.3374 (3)	0.0304 (16)	0.478 (5)
H69A	0.1370	0.6743	0.3169	0.046*	0.478 (5)
H69B	0.2045	0.5516	0.3444	0.046*	0.478 (5)
H69C	0.1024	0.6164	0.3691	0.046*	0.478 (5)

### Atomic displacement parameters (Å^2^)

**Table:**

	*U*^11^	*U*^22^	*U*^33^	*U*^12^	*U*^13^	*U*^23^
O1	0.0363 (13)	0.0429 (14)	0.0266 (13)	0.0155 (12)	0.0001 (10)	0.0051 (10)
O2	0.0213 (10)	0.0284 (11)	0.0208 (11)	−0.0039 (9)	−0.0014 (8)	0.0040 (9)
O3	0.0254 (11)	0.0656 (19)	0.0363 (14)	−0.0155 (12)	−0.0068 (10)	0.0265 (13)
O4	0.0387 (13)	0.0425 (15)	0.0340 (14)	−0.0010 (12)	0.0082 (10)	−0.0162 (12)
O5	0.0371 (13)	0.0304 (12)	0.0361 (14)	−0.0064 (11)	0.0060 (10)	−0.0111 (11)
O6	0.0366 (12)	0.0269 (12)	0.0304 (12)	−0.0036 (11)	−0.0037 (10)	−0.0030 (10)
O7	0.0407 (13)	0.0297 (13)	0.0307 (13)	0.0034 (11)	0.0017 (10)	−0.0055 (10)
O8	0.0249 (11)	0.0442 (14)	0.0213 (11)	0.0112 (10)	−0.0004 (8)	−0.0018 (10)
O9	0.0253 (10)	0.0310 (12)	0.0175 (10)	−0.0014 (10)	−0.0055 (8)	0.0018 (9)
O10	0.0259 (11)	0.0428 (13)	0.0172 (11)	0.0039 (10)	0.0034 (8)	−0.0004 (10)
O11	0.0388 (12)	0.0294 (12)	0.0207 (11)	−0.0006 (10)	0.0074 (9)	−0.0050 (9)
O12	0.0261 (10)	0.0260 (11)	0.0244 (11)	−0.0060 (9)	0.0068 (8)	−0.0036 (9)
O13	0.0405 (13)	0.0311 (12)	0.0271 (12)	−0.0126 (11)	0.0110 (10)	−0.0063 (10)
O14	0.0284 (12)	0.0488 (16)	0.0386 (14)	0.0029 (12)	−0.0063 (10)	0.0109 (12)
O15	0.0224 (11)	0.0519 (16)	0.0297 (12)	−0.0015 (11)	−0.0033 (9)	0.0046 (11)
O16	0.0275 (11)	0.0417 (13)	0.0235 (12)	−0.0037 (11)	−0.0026 (9)	0.0051 (10)
O17	0.0359 (12)	0.0297 (12)	0.0229 (11)	0.0067 (10)	0.0019 (9)	0.0017 (10)
O18	0.0383 (12)	0.0229 (11)	0.0221 (11)	0.0075 (10)	0.0099 (9)	0.0011 (9)
O19	0.0262 (10)	0.0305 (11)	0.0155 (10)	0.0048 (9)	0.0063 (8)	0.0044 (9)
O20	0.0516 (14)	0.0228 (11)	0.0225 (11)	0.0046 (11)	0.0091 (10)	0.0051 (9)
N1	0.0249 (13)	0.0355 (16)	0.0239 (14)	−0.0089 (12)	0.0031 (10)	0.0049 (11)
N2	0.0196 (12)	0.0315 (14)	0.0170 (12)	−0.0044 (11)	−0.0013 (9)	0.0077 (11)
N3	0.0254 (12)	0.0260 (14)	0.0231 (13)	−0.0092 (12)	0.0013 (10)	0.0005 (10)
N4	0.0240 (12)	0.0241 (13)	0.0161 (12)	−0.0037 (11)	0.0043 (9)	−0.0018 (10)
C1	0.0304 (17)	0.046 (2)	0.0301 (18)	0.0113 (16)	−0.0041 (13)	−0.0047 (16)
C2	0.0342 (16)	0.0246 (15)	0.0193 (15)	0.0072 (14)	0.0015 (12)	−0.0019 (12)
C3	0.0482 (19)	0.0238 (16)	0.0170 (15)	0.0076 (15)	0.0075 (13)	0.0025 (12)
C4	0.0441 (18)	0.0257 (17)	0.0188 (15)	−0.0033 (15)	0.0088 (13)	0.0027 (12)
C5	0.0305 (15)	0.0228 (15)	0.0197 (15)	−0.0030 (13)	0.0039 (12)	0.0003 (12)
C6	0.0257 (15)	0.0341 (17)	0.0186 (15)	−0.0069 (14)	0.0016 (11)	0.0053 (13)
C7	0.0228 (14)	0.0240 (15)	0.0181 (14)	−0.0034 (13)	0.0022 (11)	0.0013 (11)
C8	0.0291 (15)	0.0165 (13)	0.0149 (13)	−0.0001 (12)	0.0041 (11)	−0.0022 (11)
C9	0.0262 (14)	0.0200 (15)	0.0237 (15)	0.0009 (13)	0.0042 (12)	−0.0033 (12)
C10	0.0197 (13)	0.0250 (15)	0.0161 (13)	−0.0026 (12)	0.0032 (10)	−0.0014 (11)
C11	0.0256 (14)	0.0327 (17)	0.0164 (14)	−0.0040 (13)	−0.0014 (11)	0.0047 (12)
C12	0.0220 (14)	0.0313 (16)	0.0174 (14)	−0.0017 (13)	−0.0018 (11)	0.0056 (12)
C13	0.0226 (14)	0.0270 (16)	0.0176 (14)	−0.0034 (12)	−0.0034 (11)	0.0043 (12)
C14	0.0326 (16)	0.0294 (17)	0.0283 (17)	−0.0092 (14)	−0.0035 (13)	0.0062 (14)
C15	0.0291 (15)	0.0239 (15)	0.0261 (16)	0.0025 (14)	−0.0048 (12)	−0.0012 (13)
C16	0.0217 (13)	0.0251 (15)	0.0158 (14)	−0.0011 (12)	−0.0021 (11)	0.0003 (11)
C17	0.0186 (13)	0.0243 (15)	0.0155 (13)	0.0008 (12)	0.0011 (10)	−0.0017 (11)
C18	0.0214 (13)	0.0237 (15)	0.0171 (14)	0.0025 (12)	−0.0004 (11)	0.0012 (11)
C19	0.0228 (14)	0.0256 (16)	0.0228 (15)	0.0012 (13)	0.0034 (11)	0.0012 (12)
C20	0.0238 (14)	0.0262 (15)	0.0257 (16)	0.0005 (13)	0.0007 (12)	−0.0015 (12)
C21	0.0429 (19)	0.0313 (18)	0.0336 (19)	−0.0021 (16)	0.0002 (15)	−0.0104 (15)
C22	0.0292 (16)	0.0317 (18)	0.0227 (16)	0.0057 (14)	0.0023 (12)	−0.0073 (13)
C23	0.0262 (15)	0.0341 (18)	0.0185 (15)	0.0001 (14)	0.0059 (11)	0.0024 (13)
C24	0.0209 (13)	0.0307 (16)	0.0146 (13)	0.0008 (13)	−0.0001 (10)	0.0013 (12)
C25	0.0225 (13)	0.0193 (14)	0.0165 (13)	0.0008 (12)	−0.0006 (10)	0.0010 (11)
C26	0.0221 (13)	0.0251 (15)	0.0203 (14)	0.0006 (12)	0.0021 (11)	0.0021 (12)
C27	0.0190 (13)	0.0268 (16)	0.0214 (14)	0.0001 (12)	0.0003 (11)	0.0016 (12)
C28	0.0242 (14)	0.0257 (15)	0.0189 (14)	0.0008 (13)	−0.0036 (11)	0.0006 (12)
C29	0.0241 (14)	0.0241 (15)	0.0161 (13)	0.0006 (13)	0.0039 (10)	−0.0029 (11)
C30	0.0195 (13)	0.0256 (16)	0.0222 (15)	0.0008 (12)	−0.0006 (11)	−0.0008 (12)
C31	0.0315 (18)	0.065 (3)	0.0295 (18)	0.0187 (19)	0.0044 (14)	−0.0022 (18)
C32	0.0369 (18)	0.0341 (19)	0.0296 (18)	−0.0012 (15)	−0.0102 (14)	0.0079 (14)
C33	0.0264 (16)	0.060 (2)	0.0233 (17)	0.0033 (17)	0.0080 (12)	−0.0068 (16)
C34	0.0459 (19)	0.0319 (17)	0.0177 (15)	0.0054 (16)	0.0036 (13)	−0.0037 (13)
C35	0.0236 (14)	0.0237 (15)	0.0211 (15)	0.0034 (13)	0.0020 (11)	−0.0009 (12)
C36	0.0262 (15)	0.0294 (17)	0.0221 (15)	0.0008 (13)	0.0067 (12)	0.0020 (13)
C37	0.0271 (15)	0.0299 (16)	0.0244 (16)	−0.0056 (14)	0.0078 (12)	0.0037 (13)
C38	0.0220 (14)	0.0267 (15)	0.0217 (15)	−0.0021 (12)	0.0019 (11)	0.0022 (12)
C39	0.0203 (13)	0.0276 (16)	0.0177 (14)	−0.0021 (12)	0.0003 (10)	0.0015 (12)
C40	0.0208 (13)	0.0240 (15)	0.0168 (13)	−0.0014 (12)	−0.0014 (10)	0.0012 (11)
C41	0.0156 (12)	0.0231 (15)	0.0204 (14)	0.0018 (11)	0.0025 (10)	0.0019 (11)
C42	0.0180 (13)	0.0259 (15)	0.0202 (15)	0.0014 (12)	0.0019 (10)	0.0015 (12)
C43	0.0217 (13)	0.0247 (15)	0.0156 (13)	−0.0017 (12)	0.0021 (10)	−0.0007 (11)
C44	0.0225 (13)	0.0266 (15)	0.0154 (13)	−0.0004 (13)	0.0009 (10)	−0.0002 (12)
C45	0.0261 (14)	0.0243 (15)	0.0158 (14)	−0.0039 (12)	0.0063 (11)	−0.0011 (11)
C46	0.0248 (14)	0.0271 (15)	0.0152 (14)	−0.0017 (13)	0.0054 (11)	0.0000 (11)
C47	0.0296 (16)	0.0368 (18)	0.0199 (15)	−0.0076 (15)	−0.0001 (12)	0.0021 (13)
C48	0.0251 (14)	0.0316 (18)	0.0200 (15)	0.0035 (13)	0.0041 (11)	−0.0053 (13)
C49	0.0214 (14)	0.0276 (16)	0.0159 (14)	0.0001 (12)	0.0019 (11)	−0.0037 (11)
C50	0.0255 (14)	0.0217 (15)	0.0160 (14)	0.0001 (12)	0.0024 (11)	−0.0005 (11)
C51	0.0241 (14)	0.0257 (15)	0.0129 (13)	0.0001 (13)	0.0042 (11)	−0.0038 (11)
C52	0.0241 (14)	0.0275 (16)	0.0196 (15)	−0.0009 (13)	0.0059 (11)	−0.0009 (12)
C53	0.0216 (14)	0.0338 (17)	0.0220 (15)	−0.0028 (13)	0.0041 (11)	−0.0039 (13)
C54	0.045 (2)	0.072 (3)	0.096 (4)	−0.017 (2)	−0.029 (2)	0.045 (3)
C55	0.0277 (15)	0.0354 (18)	0.0212 (15)	0.0058 (14)	−0.0026 (12)	0.0018 (13)
C56	0.0309 (15)	0.0264 (16)	0.0173 (14)	−0.0005 (14)	0.0051 (11)	0.0001 (12)
C57	0.0217 (13)	0.0249 (15)	0.0143 (13)	−0.0004 (12)	0.0023 (10)	−0.0035 (11)
C58	0.0209 (13)	0.0212 (14)	0.0189 (14)	−0.0041 (12)	0.0029 (11)	−0.0007 (11)
C59	0.0251 (14)	0.0201 (14)	0.0193 (14)	−0.0004 (12)	0.0010 (11)	0.0020 (11)
C60	0.0169 (12)	0.0225 (15)	0.0196 (14)	0.0013 (12)	0.0018 (10)	−0.0039 (12)
C61	0.0188 (13)	0.0277 (15)	0.0159 (13)	−0.0012 (12)	0.0036 (10)	0.0002 (12)
C62	0.0235 (14)	0.0200 (14)	0.0200 (14)	0.0000 (12)	0.0012 (11)	0.0048 (12)
C63	0.0264 (15)	0.0207 (14)	0.0234 (15)	0.0010 (13)	0.0024 (11)	−0.0017 (12)
C64	0.0374 (17)	0.0230 (16)	0.0319 (18)	0.0067 (14)	0.0108 (14)	0.0035 (13)
C65	0.0361 (18)	0.099 (4)	0.0131 (16)	0.017 (2)	−0.0003 (13)	−0.0042 (19)
C66	0.0288 (16)	0.0245 (16)	0.0369 (19)	0.0046 (14)	0.0040 (13)	0.0034 (14)
O21	0.0418 (16)	0.083 (3)	0.109 (3)	−0.0281 (18)	−0.0256 (17)	0.069 (2)
C67	0.051 (2)	0.062 (3)	0.060 (3)	−0.007 (2)	−0.011 (2)	0.027 (2)
O22	0.095 (5)	0.017 (2)	0.034 (3)	−0.002 (3)	0.022 (3)	−0.0010 (19)
C68	0.141 (10)	0.025 (4)	0.031 (4)	−0.021 (5)	0.019 (5)	−0.005 (3)
O23	0.0067 (19)	0.039 (3)	0.053 (3)	0.0004 (19)	0.0034 (19)	0.008 (2)
C69	0.022 (3)	0.040 (4)	0.030 (4)	0.005 (3)	0.005 (3)	−0.009 (3)

### Geometric parameters (Å, °)

**Table:**

O1—C2	1.359 (4)	C26—C27	1.400 (4)
O1—C1	1.426 (5)	C26—H26	0.9500
O2—C10	1.236 (3)	C27—C28	1.397 (4)
O3—C11	1.223 (4)	C28—C29	1.390 (4)
O4—C22	1.384 (4)	C29—C30	1.393 (4)
O4—C21	1.428 (4)	C30—H30	0.9500
O5—C20	1.377 (4)	C31—H31A	0.9800
O5—C21	1.435 (4)	C31—H31B	0.9800
O6—C15	1.356 (4)	C31—H31C	0.9800
O6—C14	1.470 (4)	C32—H32A	0.9800
O7—C15	1.212 (4)	C32—H32B	0.9800
O8—C27	1.370 (4)	C32—H32C	0.9800
O8—C31	1.429 (4)	C33—H33A	0.9800
O9—C28	1.392 (3)	C33—H33B	0.9800
O9—C32	1.446 (4)	C33—H33C	0.9800
O10—C29	1.369 (3)	C34—H34A	0.9800
O10—C33	1.428 (4)	C34—H34B	0.9800
O11—C35	1.379 (4)	C34—H34C	0.9800
O11—C34	1.432 (4)	C35—C42	1.386 (4)
O12—C43	1.244 (4)	C35—C36	1.405 (4)
O13—C44	1.226 (4)	C36—C37	1.386 (5)
O14—C55	1.380 (4)	C36—H36	0.9500
O14—C54	1.425 (5)	C37—C38	1.401 (4)
O15—C53	1.373 (4)	C37—H37	0.9500
O15—C54	1.417 (5)	C38—C41	1.404 (4)
O16—C48	1.372 (4)	C39—C40	1.397 (4)
O16—C47	1.460 (4)	C39—H39	0.9500
O17—C48	1.198 (4)	C40—C43	1.430 (4)
O18—C60	1.373 (4)	C40—C41	1.455 (4)
O18—C64	1.423 (4)	C41—C42	1.404 (4)
O19—C61	1.385 (3)	C42—H42	0.9500
O19—C65	1.431 (4)	C43—C44	1.551 (4)
O20—C62	1.372 (4)	C45—C57	1.530 (4)
O20—C66	1.429 (4)	C45—C46	1.532 (4)
N1—C6	1.339 (4)	C45—H45	1.0000
N1—C5	1.391 (4)	C46—C47	1.516 (4)
N1—H1	0.92 (4)	C46—C49	1.520 (4)
N2—C11	1.349 (4)	C46—H46	1.0000
N2—C12	1.467 (4)	C47—H47A	0.9900
N2—H2	1.00 (4)	C47—H47B	0.9900
N3—C39	1.345 (4)	C48—C49	1.512 (4)
N3—C38	1.386 (4)	C49—C50	1.530 (4)
N3—H3A	0.91 (4)	C49—H49	1.0000
N4—C44	1.344 (4)	C50—C58	1.527 (4)
N4—C45	1.471 (4)	C50—C51	1.528 (4)
N4—H4A	0.87 (4)	C50—H50	1.0000
C1—H1A	0.9800	C51—C57	1.399 (4)
C1—H1B	0.9800	C51—C52	1.416 (4)
C1—H1C	0.9800	C52—C53	1.361 (4)
C2—C3	1.398 (5)	C52—H52	0.9500
C2—C9	1.402 (4)	C53—C55	1.395 (5)
C3—C4	1.386 (5)	C54—H54A	0.9900
C3—H3	0.9500	C54—H54B	0.9900
C4—C5	1.388 (4)	C55—C56	1.362 (4)
C4—H4	0.9500	C56—C57	1.423 (4)
C5—C8	1.402 (4)	C56—H56	0.9500
C6—C7	1.406 (4)	C58—C59	1.397 (4)
C6—H6	0.9500	C58—C63	1.400 (4)
C7—C10	1.421 (4)	C59—C60	1.391 (4)
C7—C8	1.453 (4)	C59—H59	0.9500
C8—C9	1.407 (4)	C60—C61	1.397 (4)
C9—H9	0.9500	C61—C62	1.393 (4)
C10—C11	1.543 (4)	C62—C63	1.393 (4)
C12—C24	1.525 (5)	C63—H63	0.9500
C12—C13	1.534 (4)	C64—H64A	0.9800
C12—H12	1.0000	C64—H64B	0.9800
C13—C16	1.517 (4)	C64—H64C	0.9800
C13—C14	1.531 (5)	C65—H65A	0.9800
C13—H13	1.0000	C65—H65B	0.9800
C14—H14A	0.9900	C65—H65C	0.9800
C14—H14B	0.9900	C66—H66A	0.9800
C15—C16	1.506 (4)	C66—H66B	0.9800
C16—C17	1.529 (4)	C66—H66C	0.9800
C16—H16	1.0000	O21—C67	1.411 (6)
C17—C18	1.524 (4)	O21—H21	0.8400
C17—C25	1.528 (4)	C67—H67A	0.9800
C17—H17	1.0000	C67—H67B	0.9800
C18—C19	1.408 (4)	C67—H67C	0.9800
C18—C24	1.412 (4)	O22—C68	1.405 (7)
C19—C20	1.382 (4)	O22—H22	0.8400
C19—H19	0.9500	C68—H68A	0.9800
C20—C22	1.372 (5)	C68—H68B	0.9800
C21—H21A	0.9900	C68—H68C	0.9800
C21—H21B	0.9900	O23—C69	1.428 (7)
C22—C23	1.361 (5)	O23—H23A	0.8400
C23—C24	1.406 (4)	C69—H69A	0.9800
C23—H23	0.9500	C69—H69B	0.9800
C25—C30	1.391 (4)	C69—H69C	0.9800
C25—C26	1.395 (4)		
			
C2—O1—C1	117.7 (3)	H33A—C33—H33C	109.5
C22—O4—C21	104.3 (2)	H33B—C33—H33C	109.5
C20—O5—C21	103.9 (3)	O11—C34—H34A	109.5
C15—O6—C14	110.2 (2)	O11—C34—H34B	109.5
C27—O8—C31	117.2 (2)	H34A—C34—H34B	109.5
C28—O9—C32	112.6 (2)	O11—C34—H34C	109.5
C29—O10—C33	116.6 (2)	H34A—C34—H34C	109.5
C35—O11—C34	118.0 (3)	H34B—C34—H34C	109.5
C55—O14—C54	104.8 (3)	O11—C35—C42	114.7 (3)
C53—O15—C54	104.9 (3)	O11—C35—C36	123.3 (3)
C48—O16—C47	110.9 (2)	C42—C35—C36	122.0 (3)
C60—O18—C64	115.9 (2)	C37—C36—C35	120.3 (3)
C61—O19—C65	114.4 (2)	C37—C36—H36	119.9
C62—O20—C66	116.6 (2)	C35—C36—H36	119.9
C6—N1—C5	110.1 (3)	C36—C37—C38	117.9 (3)
C6—N1—H1	130 (2)	C36—C37—H37	121.0
C5—N1—H1	120 (2)	C38—C37—H37	121.0
C11—N2—C12	120.9 (2)	N3—C38—C37	129.8 (3)
C11—N2—H2	121 (2)	N3—C38—C41	107.9 (2)
C12—N2—H2	118 (2)	C37—C38—C41	122.1 (3)
C39—N3—C38	109.7 (3)	N3—C39—C40	110.1 (3)
C39—N3—H3A	111 (2)	N3—C39—H39	125.0
C38—N3—H3A	138 (2)	C40—C39—H39	125.0
C44—N4—C45	122.5 (3)	C39—C40—C43	129.3 (3)
C44—N4—H4A	121 (2)	C39—C40—C41	105.7 (3)
C45—N4—H4A	116 (2)	C43—C40—C41	124.9 (3)
O1—C1—H1A	109.5	C38—C41—C42	119.4 (3)
O1—C1—H1B	109.5	C38—C41—C40	106.6 (3)
H1A—C1—H1B	109.5	C42—C41—C40	133.9 (3)
O1—C1—H1C	109.5	C35—C42—C41	118.2 (3)
H1A—C1—H1C	109.5	C35—C42—H42	120.9
H1B—C1—H1C	109.5	C41—C42—H42	120.9
O1—C2—C3	114.1 (3)	O12—C43—C40	122.7 (3)
O1—C2—C9	123.8 (3)	O12—C43—C44	117.6 (3)
C3—C2—C9	122.1 (3)	C40—C43—C44	119.7 (3)
C4—C3—C2	120.9 (3)	O13—C44—N4	125.0 (3)
C4—C3—H3	119.6	O13—C44—C43	122.4 (3)
C2—C3—H3	119.6	N4—C44—C43	112.7 (3)
C3—C4—C5	117.4 (3)	N4—C45—C57	108.6 (2)
C3—C4—H4	121.3	N4—C45—C46	112.6 (2)
C5—C4—H4	121.3	C57—C45—C46	108.7 (2)
C4—C5—N1	129.6 (3)	N4—C45—H45	109.0
C4—C5—C8	122.6 (3)	C57—C45—H45	109.0
N1—C5—C8	107.8 (3)	C46—C45—H45	109.0
N1—C6—C7	109.6 (3)	C47—C46—C49	102.4 (3)
N1—C6—H6	125.2	C47—C46—C45	119.3 (2)
C7—C6—H6	125.2	C49—C46—C45	109.2 (2)
C6—C7—C10	128.1 (3)	C47—C46—H46	108.5
C6—C7—C8	105.9 (3)	C49—C46—H46	108.5
C10—C7—C8	125.3 (3)	C45—C46—H46	108.5
C5—C8—C9	120.0 (3)	O16—C47—C46	104.9 (2)
C5—C8—C7	106.5 (3)	O16—C47—H47A	110.8
C9—C8—C7	133.4 (3)	C46—C47—H47A	110.8
C2—C9—C8	116.9 (3)	O16—C47—H47B	110.8
C2—C9—H9	121.6	C46—C47—H47B	110.8
C8—C9—H9	121.6	H47A—C47—H47B	108.8
O2—C10—C7	123.7 (3)	O17—C48—O16	121.8 (3)
O2—C10—C11	117.3 (3)	O17—C48—C49	129.8 (3)
C7—C10—C11	119.0 (2)	O16—C48—C49	108.1 (3)
O3—C11—N2	124.1 (3)	C48—C49—C46	103.5 (2)
O3—C11—C10	123.7 (3)	C48—C49—C50	121.5 (3)
N2—C11—C10	112.1 (2)	C46—C49—C50	113.0 (3)
N2—C12—C24	108.4 (2)	C48—C49—H49	105.9
N2—C12—C13	112.7 (3)	C46—C49—H49	105.9
C24—C12—C13	111.3 (2)	C50—C49—H49	105.9
N2—C12—H12	108.1	C58—C50—C51	112.5 (2)
C24—C12—H12	108.1	C58—C50—C49	115.1 (2)
C13—C12—H12	108.1	C51—C50—C49	108.4 (2)
C16—C13—C14	101.3 (3)	C58—C50—H50	106.8
C16—C13—C12	110.9 (2)	C51—C50—H50	106.8
C14—C13—C12	120.1 (3)	C49—C50—H50	106.8
C16—C13—H13	108.0	C57—C51—C52	119.5 (3)
C14—C13—H13	108.0	C57—C51—C50	123.8 (3)
C12—C13—H13	108.0	C52—C51—C50	116.7 (3)
O6—C14—C13	103.3 (3)	C53—C52—C51	118.4 (3)
O6—C14—H14A	111.1	C53—C52—H52	120.8
C13—C14—H14A	111.1	C51—C52—H52	120.8
O6—C14—H14B	111.1	C52—C53—O15	128.4 (3)
C13—C14—H14B	111.1	C52—C53—C55	121.6 (3)
H14A—C14—H14B	109.1	O15—C53—C55	110.0 (3)
O7—C15—O6	121.2 (3)	O15—C54—O14	109.4 (3)
O7—C15—C16	129.7 (3)	O15—C54—H54A	109.8
O6—C15—C16	109.1 (3)	O14—C54—H54A	109.8
C15—C16—C13	102.8 (2)	O15—C54—H54B	109.8
C15—C16—C17	120.6 (2)	O14—C54—H54B	109.8
C13—C16—C17	112.3 (2)	H54A—C54—H54B	108.3
C15—C16—H16	106.8	C56—C55—O14	128.6 (3)
C13—C16—H16	106.8	C56—C55—C53	122.2 (3)
C17—C16—H16	106.8	O14—C55—C53	109.2 (3)
C18—C17—C25	111.9 (2)	C55—C56—C57	117.0 (3)
C18—C17—C16	107.2 (2)	C55—C56—H56	121.5
C25—C17—C16	113.9 (2)	C57—C56—H56	121.5
C18—C17—H17	107.9	C51—C57—C56	121.2 (3)
C25—C17—H17	107.9	C51—C57—C45	122.6 (3)
C16—C17—H17	107.9	C56—C57—C45	116.1 (3)
C19—C18—C24	119.8 (3)	C59—C58—C63	119.2 (3)
C19—C18—C17	117.5 (3)	C59—C58—C50	121.5 (3)
C24—C18—C17	122.7 (3)	C63—C58—C50	119.3 (3)
C20—C19—C18	117.6 (3)	C60—C59—C58	120.4 (3)
C20—C19—H19	121.2	C60—C59—H59	119.8
C18—C19—H19	121.2	C58—C59—H59	119.8
C22—C20—O5	110.3 (3)	O18—C60—C59	124.5 (3)
C22—C20—C19	121.9 (3)	O18—C60—C61	115.1 (2)
O5—C20—C19	127.8 (3)	C59—C60—C61	120.4 (3)
O4—C21—O5	107.1 (3)	O19—C61—C62	120.4 (3)
O4—C21—H21A	110.3	O19—C61—C60	120.2 (3)
O5—C21—H21A	110.3	C62—C61—C60	119.3 (3)
O4—C21—H21B	110.3	O20—C62—C63	124.8 (3)
O5—C21—H21B	110.3	O20—C62—C61	114.7 (3)
H21A—C21—H21B	108.6	C63—C62—C61	120.5 (3)
C23—C22—C20	122.2 (3)	C62—C63—C58	120.2 (3)
C23—C22—O4	128.6 (3)	C62—C63—H63	119.9
C20—C22—O4	109.1 (3)	C58—C63—H63	119.9
C22—C23—C24	117.8 (3)	O18—C64—H64A	109.5
C22—C23—H23	121.1	O18—C64—H64B	109.5
C24—C23—H23	121.1	H64A—C64—H64B	109.5
C23—C24—C18	120.6 (3)	O18—C64—H64C	109.5
C23—C24—C12	116.2 (3)	H64A—C64—H64C	109.5
C18—C24—C12	123.0 (3)	H64B—C64—H64C	109.5
C30—C25—C26	120.2 (3)	O19—C65—H65A	109.5
C30—C25—C17	118.7 (3)	O19—C65—H65B	109.5
C26—C25—C17	121.1 (3)	H65A—C65—H65B	109.5
C25—C26—C27	119.9 (3)	O19—C65—H65C	109.5
C25—C26—H26	120.1	H65A—C65—H65C	109.5
C27—C26—H26	120.1	H65B—C65—H65C	109.5
O8—C27—C28	115.8 (3)	O20—C66—H66A	109.5
O8—C27—C26	124.4 (3)	O20—C66—H66B	109.5
C28—C27—C26	119.7 (3)	H66A—C66—H66B	109.5
C29—C28—O9	119.7 (3)	O20—C66—H66C	109.5
C29—C28—C27	120.1 (3)	H66A—C66—H66C	109.5
O9—C28—C27	120.2 (3)	H66B—C66—H66C	109.5
O10—C29—C28	114.9 (3)	C67—O21—H21	109.5
O10—C29—C30	124.9 (3)	O21—C67—H67A	109.5
C28—C29—C30	120.1 (3)	O21—C67—H67B	109.5
C25—C30—C29	120.0 (3)	H67A—C67—H67B	109.5
C25—C30—H30	120.0	O21—C67—H67C	109.5
C29—C30—H30	120.0	H67A—C67—H67C	109.5
O8—C31—H31A	109.5	H67B—C67—H67C	109.5
O8—C31—H31B	109.5	C68—O22—H22	109.5
H31A—C31—H31B	109.5	O22—C68—H68A	109.5
O8—C31—H31C	109.5	O22—C68—H68B	109.5
H31A—C31—H31C	109.5	H68A—C68—H68B	109.5
H31B—C31—H31C	109.5	O22—C68—H68C	109.5
O9—C32—H32A	109.5	H68A—C68—H68C	109.5
O9—C32—H32B	109.5	H68B—C68—H68C	109.5
H32A—C32—H32B	109.5	C69—O23—H23A	109.5
O9—C32—H32C	109.5	O23—C69—H69A	109.5
H32A—C32—H32C	109.5	O23—C69—H69B	109.5
H32B—C32—H32C	109.5	H69A—C69—H69B	109.5
O10—C33—H33A	109.5	O23—C69—H69C	109.5
O10—C33—H33B	109.5	H69A—C69—H69C	109.5
H33A—C33—H33B	109.5	H69B—C69—H69C	109.5
O10—C33—H33C	109.5		
			
C1—O1—C2—C3	179.9 (3)	C34—O11—C35—C42	167.5 (3)
C1—O1—C2—C9	1.9 (5)	C34—O11—C35—C36	−13.8 (4)
O1—C2—C3—C4	−176.4 (3)	O11—C35—C36—C37	−179.5 (3)
C9—C2—C3—C4	1.7 (5)	C42—C35—C36—C37	−0.9 (5)
C2—C3—C4—C5	−0.8 (5)	C35—C36—C37—C38	−0.7 (5)
C3—C4—C5—N1	179.4 (3)	C39—N3—C38—C37	174.3 (3)
C3—C4—C5—C8	−0.9 (5)	C39—N3—C38—C41	−1.1 (3)
C6—N1—C5—C4	179.1 (3)	C36—C37—C38—N3	−174.6 (3)
C6—N1—C5—C8	−0.6 (4)	C36—C37—C38—C41	0.2 (5)
C5—N1—C6—C7	−0.4 (4)	C38—N3—C39—C40	0.9 (3)
N1—C6—C7—C10	−170.1 (3)	N3—C39—C40—C43	−176.5 (3)
N1—C6—C7—C8	1.2 (4)	N3—C39—C40—C41	−0.3 (3)
C4—C5—C8—C9	1.6 (5)	N3—C38—C41—C42	177.7 (3)
N1—C5—C8—C9	−178.7 (3)	C37—C38—C41—C42	1.9 (4)
C4—C5—C8—C7	−178.4 (3)	N3—C38—C41—C40	0.9 (3)
N1—C5—C8—C7	1.3 (3)	C37—C38—C41—C40	−174.9 (3)
C6—C7—C8—C5	−1.5 (3)	C39—C40—C41—C38	−0.4 (3)
C10—C7—C8—C5	170.1 (3)	C43—C40—C41—C38	176.1 (3)
C6—C7—C8—C9	178.4 (3)	C39—C40—C41—C42	−176.6 (3)
C10—C7—C8—C9	−9.9 (5)	C43—C40—C41—C42	−0.1 (5)
O1—C2—C9—C8	176.9 (3)	O11—C35—C42—C41	−178.3 (2)
C3—C2—C9—C8	−1.0 (5)	C36—C35—C42—C41	3.0 (4)
C5—C8—C9—C2	−0.6 (4)	C38—C41—C42—C35	−3.5 (4)
C7—C8—C9—C2	179.4 (3)	C40—C41—C42—C35	172.4 (3)
C6—C7—C10—O2	164.5 (3)	C39—C40—C43—O12	−179.9 (3)
C8—C7—C10—O2	−5.2 (5)	C41—C40—C43—O12	4.6 (5)
C6—C7—C10—C11	−16.9 (5)	C39—C40—C43—C44	−0.1 (5)
C8—C7—C10—C11	173.3 (3)	C41—C40—C43—C44	−175.7 (3)
C12—N2—C11—O3	4.4 (5)	C45—N4—C44—O13	4.0 (5)
C12—N2—C11—C10	−178.7 (3)	C45—N4—C44—C43	−175.8 (2)
O2—C10—C11—O3	−173.4 (3)	O12—C43—C44—O13	−178.4 (3)
C7—C10—C11—O3	8.0 (5)	C40—C43—C44—O13	1.9 (4)
O2—C10—C11—N2	9.7 (4)	O12—C43—C44—N4	1.4 (4)
C7—C10—C11—N2	−168.9 (3)	C40—C43—C44—N4	−178.4 (3)
C11—N2—C12—C24	139.2 (3)	C44—N4—C45—C57	142.7 (3)
C11—N2—C12—C13	−97.3 (3)	C44—N4—C45—C46	−96.9 (3)
N2—C12—C13—C16	−80.5 (3)	N4—C45—C46—C47	49.4 (4)
C24—C12—C13—C16	41.5 (3)	C57—C45—C46—C47	169.7 (3)
N2—C12—C13—C14	37.2 (4)	N4—C45—C46—C49	−67.7 (3)
C24—C12—C13—C14	159.1 (3)	C57—C45—C46—C49	52.6 (3)
C15—O6—C14—C13	22.4 (3)	C48—O16—C47—C46	16.5 (3)
C16—C13—C14—O6	−34.4 (3)	C49—C46—C47—O16	−29.1 (3)
C12—C13—C14—O6	−156.9 (2)	C45—C46—C47—O16	−149.7 (3)
C14—O6—C15—O7	178.7 (3)	C47—O16—C48—O17	178.7 (3)
C14—O6—C15—C16	−0.3 (3)	C47—O16—C48—C49	3.6 (3)
O7—C15—C16—C13	159.0 (3)	O17—C48—C49—C46	163.3 (3)
O6—C15—C16—C13	−22.1 (3)	O16—C48—C49—C46	−22.1 (3)
O7—C15—C16—C17	33.1 (5)	O17—C48—C49—C50	35.2 (5)
O6—C15—C16—C17	−148.0 (3)	O16—C48—C49—C50	−150.2 (3)
C14—C13—C16—C15	34.0 (3)	C47—C46—C49—C48	30.8 (3)
C12—C13—C16—C15	162.6 (2)	C45—C46—C49—C48	158.1 (2)
C14—C13—C16—C17	165.1 (2)	C47—C46—C49—C50	164.0 (2)
C12—C13—C16—C17	−66.3 (3)	C45—C46—C49—C50	−68.6 (3)
C15—C16—C17—C18	176.5 (2)	C48—C49—C50—C58	43.4 (4)
C13—C16—C17—C18	55.1 (3)	C46—C49—C50—C58	−80.4 (3)
C15—C16—C17—C25	52.3 (4)	C48—C49—C50—C51	170.5 (3)
C13—C16—C17—C25	−69.1 (3)	C46—C49—C50—C51	46.6 (3)
C25—C17—C18—C19	−79.5 (3)	C58—C50—C51—C57	112.6 (3)
C16—C17—C18—C19	155.0 (2)	C49—C50—C51—C57	−15.9 (4)
C25—C17—C18—C24	99.6 (3)	C58—C50—C51—C52	−67.4 (3)
C16—C17—C18—C24	−25.9 (4)	C49—C50—C51—C52	164.1 (3)
C24—C18—C19—C20	1.3 (4)	C57—C51—C52—C53	−0.2 (4)
C17—C18—C19—C20	−179.5 (3)	C50—C51—C52—C53	179.7 (3)
C21—O5—C20—C22	13.3 (3)	C51—C52—C53—O15	178.7 (3)
C21—O5—C20—C19	−167.6 (3)	C51—C52—C53—C55	−1.2 (5)
C18—C19—C20—C22	−1.7 (4)	C54—O15—C53—C52	−172.0 (4)
C18—C19—C20—O5	179.3 (3)	C54—O15—C53—C55	7.9 (4)
C22—O4—C21—O5	22.7 (3)	C53—O15—C54—O14	−13.2 (5)
C20—O5—C21—O4	−22.1 (3)	C55—O14—C54—O15	13.4 (5)
O5—C20—C22—C23	179.4 (3)	C54—O14—C55—C56	170.9 (4)
C19—C20—C22—C23	0.2 (5)	C54—O14—C55—C53	−8.3 (4)
O5—C20—C22—O4	0.8 (4)	C52—C53—C55—C56	0.9 (5)
C19—C20—C22—O4	−178.4 (3)	O15—C53—C55—C56	−179.0 (3)
C21—O4—C22—C23	166.9 (3)	C52—C53—C55—O14	−179.8 (3)
C21—O4—C22—C20	−14.6 (3)	O15—C53—C55—O14	0.3 (4)
C20—C22—C23—C24	1.6 (5)	O14—C55—C56—C57	−178.3 (3)
O4—C22—C23—C24	179.9 (3)	C53—C55—C56—C57	0.8 (5)
C22—C23—C24—C18	−1.9 (4)	C52—C51—C57—C56	1.9 (4)
C22—C23—C24—C12	173.4 (3)	C50—C51—C57—C56	−178.0 (3)
C19—C18—C24—C23	0.5 (4)	C52—C51—C57—C45	−174.3 (3)
C17—C18—C24—C23	−178.7 (3)	C50—C51—C57—C45	5.7 (4)
C19—C18—C24—C12	−174.5 (3)	C55—C56—C57—C51	−2.2 (4)
C17—C18—C24—C12	6.3 (4)	C55—C56—C57—C45	174.3 (3)
N2—C12—C24—C23	−64.3 (3)	N4—C45—C57—C51	98.9 (3)
C13—C12—C24—C23	171.3 (3)	C46—C45—C57—C51	−23.9 (4)
N2—C12—C24—C18	110.9 (3)	N4—C45—C57—C56	−77.5 (3)
C13—C12—C24—C18	−13.5 (4)	C46—C45—C57—C56	159.7 (3)
C18—C17—C25—C30	148.3 (3)	C51—C50—C58—C59	−34.1 (4)
C16—C17—C25—C30	−90.0 (3)	C49—C50—C58—C59	90.9 (3)
C18—C17—C25—C26	−29.8 (4)	C51—C50—C58—C63	143.9 (3)
C16—C17—C25—C26	91.9 (3)	C49—C50—C58—C63	−91.2 (3)
C30—C25—C26—C27	1.5 (4)	C63—C58—C59—C60	1.6 (4)
C17—C25—C26—C27	179.6 (3)	C50—C58—C59—C60	179.5 (3)
C31—O8—C27—C28	−175.5 (3)	C64—O18—C60—C59	1.7 (4)
C31—O8—C27—C26	2.8 (5)	C64—O18—C60—C61	−176.6 (3)
C25—C26—C27—O8	−178.6 (3)	C58—C59—C60—O18	−178.0 (3)
C25—C26—C27—C28	−0.3 (5)	C58—C59—C60—C61	0.3 (4)
C32—O9—C28—C29	−93.1 (3)	C65—O19—C61—C62	75.3 (4)
C32—O9—C28—C27	86.4 (4)	C65—O19—C61—C60	−108.7 (4)
O8—C27—C28—C29	176.7 (3)	O18—C60—C61—O19	−0.1 (4)
C26—C27—C28—C29	−1.7 (5)	C59—C60—C61—O19	−178.6 (3)
O8—C27—C28—O9	−2.7 (4)	O18—C60—C61—C62	175.9 (2)
C26—C27—C28—O9	178.8 (3)	C59—C60—C61—C62	−2.5 (4)
C33—O10—C29—C28	164.1 (3)	C66—O20—C62—C63	−31.8 (4)
C33—O10—C29—C30	−14.8 (5)	C66—O20—C62—C61	149.0 (3)
O9—C28—C29—O10	3.0 (4)	O19—C61—C62—O20	−1.9 (4)
C27—C28—C29—O10	−176.5 (3)	C60—C61—C62—O20	−177.9 (3)
O9—C28—C29—C30	−178.1 (3)	O19—C61—C62—C63	178.8 (3)
C27—C28—C29—C30	2.5 (5)	C60—C61—C62—C63	2.8 (4)
C26—C25—C30—C29	−0.8 (5)	O20—C62—C63—C58	180.0 (3)
C17—C25—C30—C29	−178.9 (3)	C61—C62—C63—C58	−0.8 (4)
O10—C29—C30—C25	177.6 (3)	C59—C58—C63—C62	−1.4 (4)
C28—C29—C30—C25	−1.3 (5)	C50—C58—C63—C62	−179.3 (3)
